# ExoMeg1: a new exonuclease from metagenomic library

**DOI:** 10.1038/srep19712

**Published:** 2016-01-27

**Authors:** Rita C. B. Silva-Portela, Fabíola M. Carvalho, Carolina P. M. Pereira, Nadja C. de Souza-Pinto, Mauro Modesti, Robert P. Fuchs, Lucymara F. Agnez-Lima

**Affiliations:** 1 Departamento de Biologia Celular e Genética, Centro de Biociências, Universidade Federal do Rio Grande do Norte, Natal, RN, 59072-970, Brasil; 2 Departamento de Bioquímica, Instituto de Química, Universidade de São Paulo (USP), São Paulo, Brasil; 3 Homologous Recombination, NHEJ and Maintenance of Genomic Integrity CNRS, UMR7258, Marseille, F-13009, France; 4 Team DNA Damage Tolerance CNRS, UMR7258, Marseille, F-13009, France; 5 Inserm, U1068, CRCM, Marseille, F-13009, France; 6 Institut Paoli-Calmettes, Marseille, F-13009, France; 7 Aix-Marseille University, UM 105, F-13284, Marseille, France

## Abstract

DNA repair mechanisms are responsible for maintaining the integrity of DNA and are essential to life. However, our knowledge of DNA repair mechanisms is based on model organisms such as *Escherichia coli,* and little is known about free living and uncultured microorganisms. In this study, a functional screening was applied in a metagenomic library with the goal of discovering new genes involved in the maintenance of genomic integrity. One clone was identified and the sequence analysis showed an open reading frame homolog to a hypothetical protein annotated as a member of the Exo_Endo_Phos superfamily. This novel enzyme shows 3′-5′ exonuclease activity on single and double strand DNA substrates and it is divalent metal-dependent, EDTA-sensitive and salt resistant. The clone carrying the hypothetical ORF was able to complement strains deficient in recombination or base excision repair, suggesting that the new enzyme may be acting on the repair of single strand breaks with 3′ blockers, which are substrates for these repair pathways. Because this is the first report of an enzyme obtained from a metagenomic approach showing exonuclease activity, it was named ExoMeg1. The metagenomic approach has proved to be a useful tool for identifying new genes of uncultured microorganisms.

In nature, microorganisms live under a variety of environmental stress conditions induced by physical or chemical agents that, in addition to byproducts of cellular metabolism, are able to generate different DNA lesions, which if not repaired may induce mutations, genetic recombination and inhibition or modification of DNA replication and/or transcription. Considering these deleterious effects of DNA damage, it is predicable that all forms of life possess specialized mechanisms to recognize and repair different DNA lesions, favoring direct or indirect approaches for their adaptation and survival^1^,^2^. In fact, considerable conservation of repair functions among different species of prokaryotes and eukaryotes has been observed, which may or may not be accompanied by corresponding conservation of protein sequence^1^,^3^,^4^. However, knowledge of DNA repair mechanisms in bacteria is based on cultured microorganisms, mainly using the model organism *Escherichia coli*^5^,^6^,^7^, which represents less than 1% of the microbial diversity estimated to be on Earth because more than 99% of microbial species have not been cultured using traditional methods^8^,^9^.

To circumvent the limitations of culture methods, the metagenomic approach, defined as collective microbial genome analysis^10^, has been developed and improved to complement culture-dependent traditional approaches. This methodology allows for the extraction of DNA from environmental samples and subsequent cloning using a cultivable host strain as well as direct shotgun sequencing by next generation sequencing strategies. Numerous studies have confirmed that access to genetic patrimony of metagenomes provides a rich source for finding new genes and for understanding biological processes and microbial ecology^11^,^12^,^13^,^14^.

Functional screens of metagenomic libraries have allowed for the discovery of new genes that encode proteins with either known or new functions because these screens are independent of sequence similarity to known genes. The selection of active clones expressing a desired phenotype is followed by sequencing and characterization by biochemical specific assays. The major advantage of this approach is that, in general, only functional gene products are identified. However, successful heterologous expression of recombinant proteins is necessary, which is a primary limitation of this approach, in addition to the lack of innovative screening methods and novel activity assays^9^,^15^,^16^ .

The study of genome stability and DNA repair mechanisms is extremely important for not only understanding molecular mechanisms and evolutionary implications but also because of medical relevance because several diseases are attributed to deficiencies in DNA repair mechanisms, as well as the resistance of microorganisms to host immune systems, which is an important aspect of the host-pathogen relationship^1^,^2^. Despite this importance, few studies are available that use the metagenomic approach for the discovery of new genes involved in the maintenance of genome integrity^13^,^17^,^18^,^19^. As such, no protein directly related to DNA repair activity has been successfully purified and biochemically characterized. In this study, a new exonuclease potentially related to DNA repair has been identified and experimentally characterized using a metagenomic approach with functional screening.

## Results

**Figure 1:**
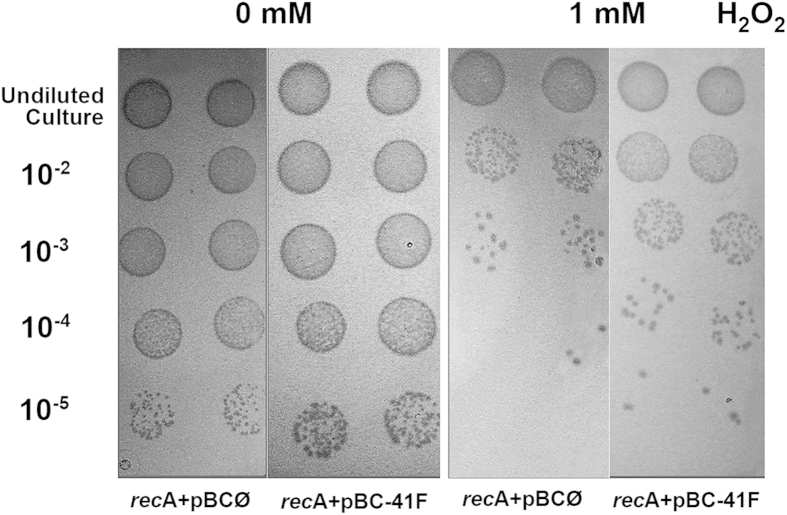
Complementation assay in the *E. coli rec*A- strain. An increase in H_2_O_2_ resistance was observed in the DH10B strain due to the presence of an environmental clone (pBC-41 F, containing the ExoMeg1 ORF). Undiluted cultures and serial dilutions (10^−2^, 10^−3^, 10^−4^ and 10^−5^) from cultures (OD_600_ of 0.8) were plated on LB agar containing 1 mM H_2_O_2_.

In this study, 2,688 clones of one environmental DNA library was screened through a complementation assay using the DH10B *E. coli* strain as host, which is deficient in the DNA repair gene *recA*, rendering it sensitive to several mutagenic agents, including ultraviolet light (UV) and hydrogen peroxide (H_2_O_2_). The screening was conducted on H_2_O_2_-LB agar plates, and one candidate clone, named pBC-41F, was pre-selected. Afterward, to avoid the isolation of a false-positive clone, the recombinant plasmid was isolated and retransformed into DH10B cells to perform a new activity confirmation assay in selective medium. Since it showed positive results in a complementation assay in the *recA-* strain (Fig. 1), it was selected for the functional characterization described in this work.

### *In silico* analysis of the pBC-41F clone

The size of the environmental insert cloned into the pBC plasmid was 1.5 kb, and sequence analysis revealed the presence of an open reading frame (ORF) of 975 bp encoding a polypeptide of 324 amino acids with a predicted molecular weight and theoretical isoelectric point (pI) of approximately 37 KDa and 5.02, respectively. The search for homologous sequences revealed the absence of significant similarity to known proteins deposited in the non-redundant database (NCBI) but showed similarity to hypothetical protein BfaeM_06119 (ZP_09858401.1, presenting 17% identity, e-value 2e^−62^ and 99% coverage) of *Bacteroidesfaecis* MAJ27, containing a conserved domain related to the Exo_Endo_Phos (PF03372) superfamily (EEP).

**Figure 2:**
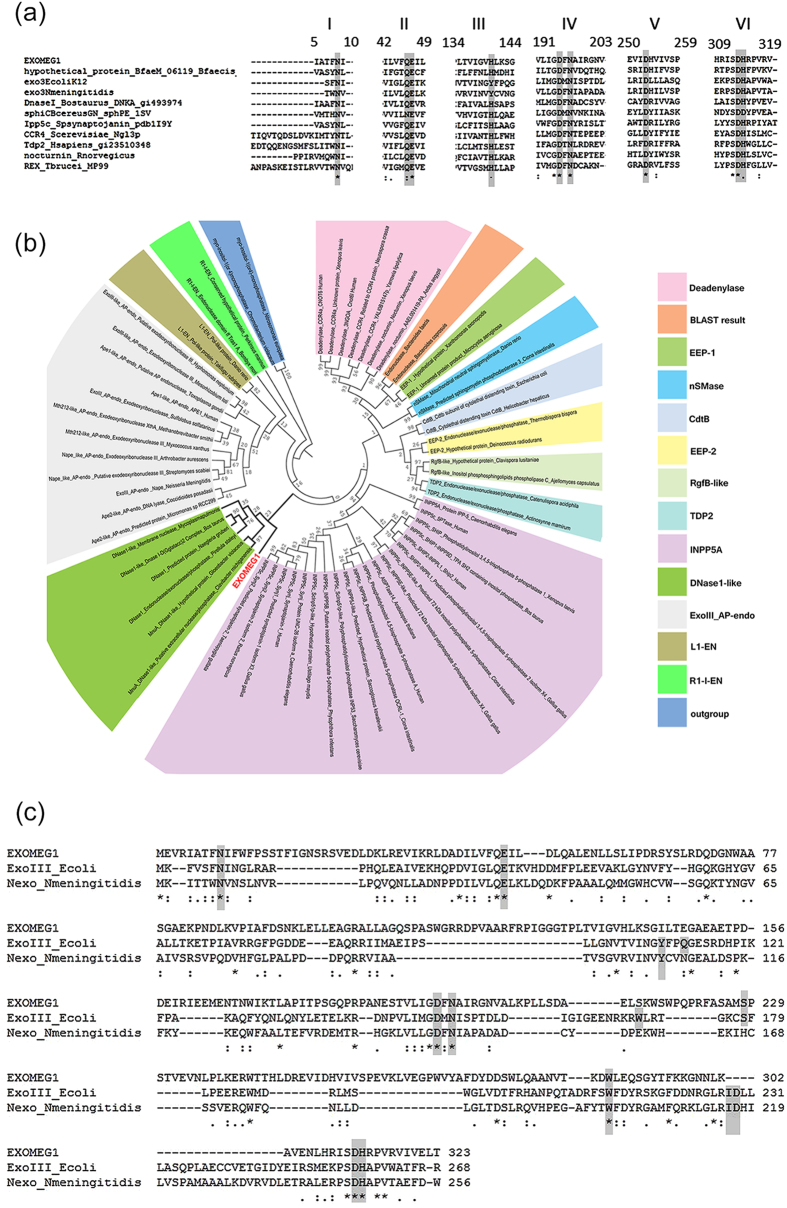
*In silico* analysis of the ExoMeg1 sequence. (**A**) Multiple sequence alignments including the metagenomic nuclease (ExoMeg1) and various EEP members. The conserved motifs are highlighted, and the regions containing the motifs are numbered above the alignment. The alignment was generated by T-coffee server and visualized by MEGA5. (**B**) Maximum likelihood of phylogenetic reconstruction of ExoMeg1 and other EEP subfamilies. ExoMeg1 is in a separate branch not grouped to any EEP subfamily members. Myo-inositol-1(or 4)-monophosphatase sequences from the IMPase-like superfamily were used as the outgroup. Bootstrap values were based on 1000 replicates. (**C**) Multiple sequence alignment including the amino acid sequences of Exonuclease III (*E. coli*), Exonuclease Nexo (*N. meningitidis*), and metagenomic nuclease ExoMeg1 described in this work. The catalytic domain in ExoIII includes Asp229, His259 and Glu34 corresponding to a pocket surrounded by Gln112, Asn153, Try109, Asn7 and Trp212. The residue Asp146 in Nexo is related to 3′ phosphodiesterase activity whereas the residues D313 and H314 in ExoMeg1 are conserved among EEP members, and they are related to 3′-5′exonuclease activity. Most of the catalytic residues required for AP endonuclease activity are present in ExoMeg1 and are highlighted in gray. The alignment was generated by T-coffee server. The numbers of the amino acid residues are given the right of each lane.

The multiple sequence alignments of metagenomic ORF against different EEP family members showed the presence of six conserved catalytic residues, which suggests that the environmental protein belongs to this superfamily and shares the same catalytic mechanism with other EEP members (Fig. 2A). This ORF was named exonuclease 1 from the metagenome, ExoMeg1, due to its exonuclease activity described below. In the phylogenetic reconstruction, ExoMeg1 is present in a monophyletic group, composed of DNase I and MnuA subfamily members. The phylogeny obtained suggests that the divergence of ExoMeg1 is an ancestral event separate from the divergence of the DNase I and MnuA sub families. Thus, ExoMeg1 is in a separate branch, not grouping with any members of EEP subfamilies (Fig. 2B), suggesting that this enzyme belongs to a new sub family.

Considering that some EEP-family members present activity as AP endonuclease/3′-phosphodiesterases important for the repair of DNA lesions generated by H_2_O_2_, such as abasic sites or 3′ blocking damages, we compared ExoMeg1 with the DNA repair enzymes exonuclease III (Xth) from *E. coli* and Nexo from *N. meningitides*, which are members of the Xth sub family. Alignment indicates the presence of conserved residues (N9, E47, I252, D195, N197, W217, S229, D253 and H314) potentially involved in AP endonuclease/3′-phosphodiesterase activities (Fig. 2C). However, the residue W212 found in exonuclease III of *E. coli* and some loops and α-helix regions important for AP-endonuclease activity are absent in ExoMeg1.

### Complementation assay in an *E. coli* strain deficient in DNA repair

**Figure 3:**
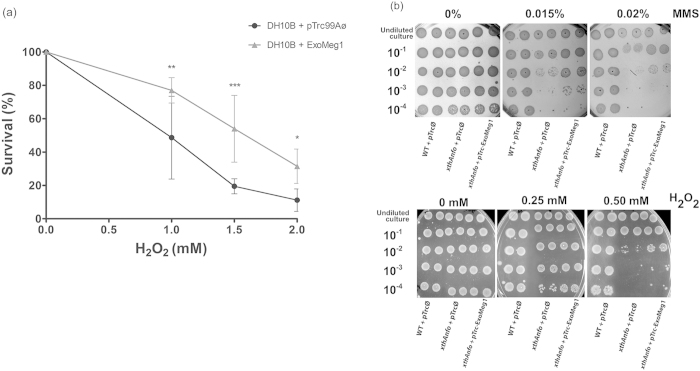
Complementation assay in the *E. coli* DNA repair deficient strains. (**A**) Survival curve of the *rec*A- strain subjected to increasing concentrations of H_2_O_2_ (0–2 mM) for 30 min. *rec*A- strain transformed with empty vector (•) and with pTrc-ExoMeg1 (▲).Values are the mean ± SD of three independent experiments in duplicate. Statistical analysis by two-way ANOVA and Bonferroni post-test, *P < 0.05, **P < 0.01 and ***P < 0.001. (**B**) Complementation assay in the *E. coli xth*A*-nfo*- strain. A survival increase in plates with MMS or H_2_O_2_ was observed in the presence of pTrc-ExoMeg1 in the *E. coli* double mutant strain. The cultures were induced by 0.1 mM IPTG. Undiluted cultures and serial dilutions (10^−1^, 10^−2^, 10^−3^ and 10^−4^) from cultures (OD_600_ of 0.8) were plated on LB agar containing different concentrations of MMS or H_2_O_2_. AB1157 was used as wild type (WT) strain proficient in DNA repair.

The recombinant plasmid containing the ExoMeg1 ORF (pTrc- ExoMeg1) was able to decrease the sensitivity of the *rec*A- strain to H_2_O_2_ because it increased survival by approximately 20% when compared to the same mutant strain transformed with the empty vector (Fig. 3A). Additionally, due to the presence of domains peculiar to the EEP family (Fig. 2A), pTrc-ExoMeg1 construction was transformed into *E. coli* strain deficient in exonuclease III (*xth*A) and endonuclease IV (*nfo*). The results show that ExoMeg1 complemented the DNA repair deficiency (*xth*A-*nfo*-), allowing for better survival after methyl methanesulfonate (MMS) or H_2_O_2_ treatments (Fig. 3B). Together, these results suggest a repair activity for ExoMeg1.

### Activity assays

**Figure 4:**
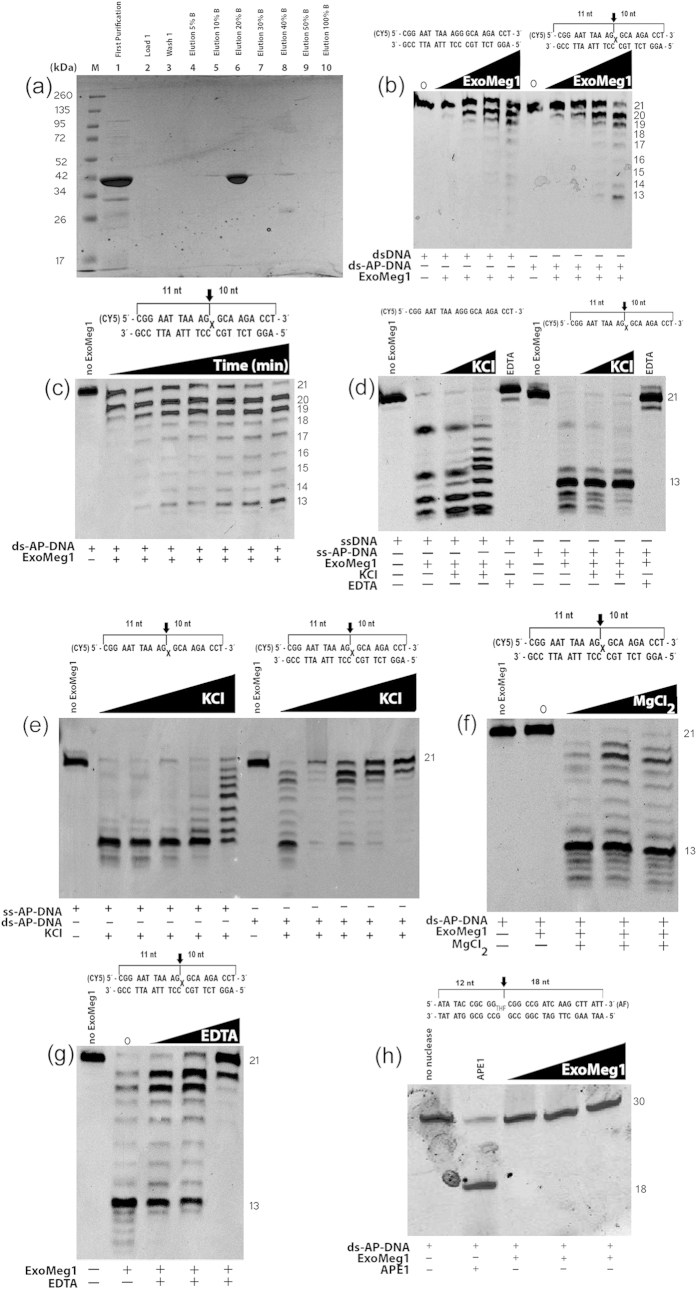
Purification and *in vitro* characterization of ExoMeg1. (**A**) SDS-PAGE analysis of metagenomic protein purification through a HiTrap Q HP. Aliquots (10 μL) of each sample were electrophoresed on a 12% SDS-PAGE, which was stained with Coomassie blue. Lane M, molecular weight marker SpectraBR; Lane 1, purification by HisTrap column; Lanes 2-10, steps of purification by HiTrap Q HP. The protein was eluted with 20% buffer B (lane 6). (**B**) Identification of 3′-5′exonuclease activity on dsDNA or ds-AP-DNA with increasing concentrations of ExoMeg1. The AP endonuclease activity should result in an 11-mer product. However, ExoMeg1 has shown only the exonuclease activity, generating a 13-mer product. (**C**) Activity assay versus time of incubation at 37 °C using ds-AP-DNA. (**D**) Identification of 3′-5′exonuclease activity on the single strand substrate and the effect of salt on the activity of ExoMeg1. Lanes 1 and 6, reactions in the absence of ExoMeg1 and KCl; Lanes 2 and 7, reactions in the presence of ExoMeg1 but absence of KCl; Lanes 3 and 8, reactions including ExoMeg1 and 50 mM KCl; Lanes 4 and 9, reactions including EXOMEG1 and 200 mM KCl; lanes 5 and 10, reactions containing 10 mM EDTA. (**E**) Analysis of salt effects on ExoMeg1 activity using increasing concentrations of KCl (50, 100, 250, 500 and 1000 mM). (**F**) Mg2 + dependence of ExoMeg1 activity. The reactions contain increasing concentrations of MgCl2 (0, 5, 10 and 15 mM). The ExoMeg1 activity is totally inhibited by the absence of MgCl2 (lane 2). (**G**) Effect of EDTA on ExoMeg1 activity. The reactions contain increasing concentrations of EDTA (0, 1, 5 and 10 mM). The progression of 3′-5′ exonuclease activity is gradually inhibited by EDTA. (**H**) AP endonuclease assay using a 30-mer labeled at the 3′ end with increasing concentrations of ExoMeg1. Commercial APE1 enzyme (NEB Biolabs) was used as the positive control (lane 2).

The environmental protein was obtained by two different and consecutive purification processes using HisTrap FF and HiTrap Q HP columns, and, for the last step, a salt gradient was used, being the purified protein eluted in 20% buffer B as verified by SDS-PAGE (Fig. 4A).

To determine whether ExoMeg1 has AP endonuclease activity, we performed *in vitro* assays using 5′-Cy5-labeled substrates consisting of 21-mer oligonucleotides containing a single abasic site at position 10. Incubation of the purified protein with the AP-containing 21-mer oligonucleotide generated a 13-nt product, which could not be observed in the reaction using the control oligonucleotide without the abasic site. However, the AP endonuclease activity should result in an 11-mer product. Additionally, progressive shortening of the full length 21-mer substrate was observed, exhibiting a ladder pattern for the fragments in the gel, indicating that ExoMeg1 degrades the dsDNA and ssDNA substrates from the 3′ end, suggesting an exonuclease activity with 3′ to 5′ polarity (Fig. 4B–G).

The effect of salt concentration and metal dependency on ExoMeg1 activity was also investigated. High salt concentration was able to affect ExoMeg1′s 3′-5′exonuclease activity in both ssDNA and dsDNA substrates (Fig. 4D,E). However, the exonuclease activity on ssDNA is more resistant to high salt concentration because the ladder pattern is still observed at higher KCl concentrations. At lower KCl concentrations, it is possible to observe the degradation of higher products generating smaller fragments, suggesting faster activity at lower salt concentrations (Fig. 4E, lane 6). Additionally, it was observed in this work that ExoMeg1 activity requires a divalent metal cofactor because the exonuclease activity was observed only in presence of magnesium (Fig. 4F). To confirm that ExoMeg1 is a metal-dependent enzyme, reactions were performed including increasing concentrations of EDTA and compared to the reaction without EDTA. From this assay, it was observed that EDTA inhibited the exonuclease activity, confirming ExoMeg1′s dependence on a divalent metal (Fig. 4F,G).

The occurrence of an 13-nt product generated by the activity of ExoMeg1 raises the question of whether this product results from cleavage of the abasic site by a non-canonical mechanism, different from other AP endonucleases, such as exonuclease III, or whether such a product would be assigned to an activity pause site of ExoMeg1. To answer this question, we conducted an activity assay using a 3′-labeled oligonucleotide containing an abasic site analogue. The data showed that ExoMeg1 has no direct AP endonuclease activity or 5′-3′exonuclease activity because no cleavage product was observed (Fig. 4H). Therefore, ExoMeg1 is a 3′-5′ exonuclease acting on ssDNA and dsDNA with ssDNA being its preferred substrate.

## Discussion

The metagenomic approach has been considered to be an efficient method for obtaining novel and useful genes from uncultured microorganisms. In this work, functional screening allowed for the identification of a metagenomic clone that shows activity that is potentially related to DNA repair because it was able to complement the DNA repair deficiency in *recA-* and *xth-nfo-* strains. Sequence analysis revealed an ORF with similarity to a hypothetical protein containing a conserved domain related to the EEP superfamily. This superfamily contains various structural and functionally related enzymes, which share a common mechanism of phosphodiester bond cleavage but act on different substrates. EEP representative enzymes include magnesium dependent endonucleases (L1-EN, DNase I, APE1, APE2, Xth), exonucleases (Xth, REX1, REX2, APE1), phosphatases (I5PP), sphingomyelinases and others proteins with unknown functions^20^,^21^,^22^,^23^,^24^,^25^.

ExoMeg1 has 3′-5′exonuclease activity, and an unexpected 13-nt product was observed when tested with an oligonucleotide containing a single abasic site. This 13-nt product can result from a pausing site of the exonuclease activity in the vicinity of the abasic site because direct AP endonuclease activity was not observed. Therefore, ExoMeg1 is a 3′-5′exonuclease that likely tends to pause near DNA damage similar to what has been observed for other 3′-5′ exonuclease enzymes^26^,^27^; however, it continues over the substrate to generate smaller products, as seen in Fig. 4.

In fact, the presence of most of the conserved residues important for AP endonuclease and 3′ phosphodiesterase activities in Xth of *E. coli* and Nexo of *N. meningitides* were found in the ExoMeg1 sequence, suggesting a DNA repair function. However, the absence of the equivalent residue to W212 found in Xth and the absence of structural elements, which produce direct contacts with nucleic acids, such as loops or α-helix that have been described to confer selectivity to the cleavage of abasic sites^28^,^29^,^30^, provide possible reasons for the lack of classic AP endonuclease activity in the ExoMeg1 protein. Similarly, it was shown that Nexo from *N. meningitidis* is a Xth family member with significant 3′ phosphodiesterase and exonuclease activity, which is fundamental in the repair of 3′ blocking damages generated by oxidative stress, and has no AP endonuclease activity^31^,^32^. The presence of these regions is crucial for AP endonuclease activity, as demonstrated by Cal *et al.*^28^, which, with addition of some amino acids of Xthα-helix, converted a bovine pancreatic DNase I to a repair endonuclease with a high selectivity for abasic sites. This specific region is absent in ExoMeg1. This also corroborates our phylogenetic analysis in which ExoMeg1 is closest to the DNAse1-like branch. These data emphasize that despite EEP members sharing the mechanism of phosphodiester bond cleavage, there are peculiarities that define the specificity of a given enzyme for different substrates^20^.

Complementation assays showed that ExoMeg1 is able to complement the *recA-* and *xth-nfo-* strains, suggesting that this enzyme is involved in the repair of DNA lesions caused by H_2_O_2_ and MMS. Both agents can induce abasic sites and strand breaks with 3′ blocking ends that are substrates for recombination or base excision repair^33^,^34^,^35^. Therefore, the 3′-5′exonuclease activity of ExoMeg1 may be acting on the repair of single strand breaks with 3′ blockers because AP endonuclease activity was not observed.

Some 3′-5′exonucleases are specialized to remove 3′ blocking damages, which are very cytotoxic if not repaired. As an example, Nexo has 3′-5′exonuclease and 3′ phosphodiesterase activities that are able to improve resistance to H_2_O_2_ and paraquat, but it has no detectable AP endonuclease activity, which is attributed to the presence of His167 instead of G or S, as found in other Xth subfamily members. Nexo is an essential enzyme specialized for 3′-end processing, mainly for removing 3′ PO_4_ blocking damages^31^,^32^ that are generated by some bi-functional glycosylases (as FPG) but can also arise directly from oxidative damage^36^,^37^,^38^. In another example, the 3′–5′ exonuclease activity of Rad1-Rad10 from *Saccharomyces cerevisiae* is required for removing 3′phosphoglycolate, a 3′ blocking lesion generated by oxidative attacks on sugar in DNA^39^. Mazouzi *et al.*^40^ showed that Xth and APE1 efficiently removed the lesion (5′*S*)-8,5′-*cyclo*-2′-deoxyadenosine (*S*-cdA) when present at 3′ termini of a recessed, nicked, or gapped DNA duplex. However, these enzymes are not able to remove the *S*-cdA adducts when present at 1 or more nt away from the 3′ end.

Other functions has also been attributed to 3′–5′ exonucleases, such as nucleotide incision repair (NIR) mediated by Nfo and in alternative pathways for repairing 8-oxoG, mismatches and 3′ blocking groups by 3′–5′exonuclease function of Apn1^41^,^42^. Likewise, human Ape2, aside from its inefficient AP endonuclease activity *in vitro*, has strong 3′-5′ exonuclease activity that is able to remove mismatched nucleotides from the 3′ prime end, suggesting a role as a proofreader in DNA repair synthesis similar to human APE1 proofreading errors by Pol-β^43^.

Salt concentration and specific metals may influence DNA cleavage by nucleases. Protein-DNA interactions are highly dependent on salt concentration, and the nuclease enzymes have conserved motifs that form active sites that coordinate catalytically essential divalent cations, such as magnesium, manganese, calcium or zinc as a cofactor^44^,^45^. In this study, we observed that ExoMeg1 is divalent metal-dependent because it is inactive in the absence of any divalent metal but is active in presence of Mg^2+^. Furthermore, it is inactivated by EDTA, a chelating agent, similarly to that described for Dnase I and Xth^45^,^46^,^47^. ExoMeg1 is a salt resistant enzyme because inhibition of the exonuclease activity was not completely achieved. The exonuclease activity was observed even with the highest tested concentration of 1 M of KCl. This concentration is approximately 10-fold higher than what was described for Xth and Nfo^38^,^48^. This high resistance of ExoMeg1 to salt concentration may reflect environmental adaptation because the metagenomic library was obtained from DNA extracted from semiarid soil exposed to environmental stresses, including salinity, drought and UV radiation^49^. These environmental stressors may lead to the generation of reactive oxygen species (ROS) and, consequently, oxidative damage of nucleic acids, including modified bases and single or double strand DNA breaks. In fact, it has been related that a high concentration of NaCl increases DNA breaks in both cell culture and *in vivo*^50^. Thus, considering that well-known exonucleases of model organisms are sensitive to high salinity is very useful a new salt-resistant exonuclease, Exomeg1, able to decrease the sensitivity to oxidative stress and thus confer an important environmental adaptation.

In conclusion, in this study, a novel protein potentially related to DNA repair, initially annotated as a hypothetical protein of unknown function, has been characterized experimentally *in vivo* and *in vitro* and it is the first enzyme obtained from the metagenomic approach showing this function. In this first study on ExoMeg1, we described its 3′–5′ exonuclease activity and its absence of AP endonuclease function. The next steps will be to test its 3′ phosphodiesterase activity and identify the probable 3′ blocking lesions that may be removed by this new enzyme. The results obtained in this work support the idea that metagenomes represent a rich source for finding new genes because it is expected that uncultivated fraction include many microorganisms distantly related to cultivated microorganisms. In fact, a vast portion of genes described in genome or metagenome projects are classified as coding hypothetical proteins. Hence, the discovery the functions of these new genes represents one of the biggest challenges of genome or metagenome projects because if there is no similarity to sequences already deposited in databanks, such as NCBI/nr or UniProt, in general, it is impossible to infer a function. Here, we were able to attribute function to a hypothetical ORF, contributing to the understanding of the diversity of the DNA repair process and generation of many new questions to be answered with new assays using various substrates and mutants of ExoMeg1.

## Methods

### Culture conditions

The *E. coli* strains and plasmids used in this study are listed in Table 1. The strains were routinely grown in Lysogeny Broth (LB) in g/L: Tryptone 10, Yeast Extract 5, NaCl 10; at 37 °C and, when necessary, were supplemented with 25 μg/mL chloramphenicol, 50 μg/mL kanamycin or 100 μg/mL ampicillin to maintain the plasmid vectors. All enzymes were purchased from New England Biolabs.

### Library construction and functional screening

Soil samples (5–10 cm) were collected from João Câmara city, located in northeast Brazil (S5° 30′ 51.81″ O35° 54′ 17:13″). This city is localized in a semiarid region, and it is included in the Caatinga biome, which is an exclusively Brazilian biome^49^. The collection was performed using sterile tubes and spatulas, and environmental DNA (eDNA) was extracted using the commercial kit PowerMax TM Soil DNA Isolation Kit (MOBIO Laboratories) from 10 g of soil. The eDNA was partially digested with the enzymes EcoRV, SmaI and PvuII, and the 1.5–2 kb fragments were purified from agarose gel and linked to the cloning vector pBC previously EcoRV-linearized and CIAP-dephosphorylated. The library obtained was transformed into the DNA repair-deficient DH10B (*rec*A-) *E. coli* strain and plated on chloramphenicol/X-gal-LB agar. For functional screening, the library was replicated on LB agar containing different concentrations of hydrogen peroxide (0–3 mM H_2_O_2_, Merck). The strain transformed with the empty vector was used as the negative control. To avoid selecting a false positive clone, the recombinant plasmid was isolated and retransformed into DH10B to confirm that the acquired phenotype was due to the presence of the environmental insert.

### ORF identification and sequence analysis

The positive clones were sequenced using a DYEnamic ET Dye Terminator Cycle Sequencing Kit for MegaBACE 500 (Amersham Biosciences) following the manufacturer′s instructions. ORF predictions were performed using the program ORF Finder available online on the website (http://www.ncbi.nlm.nih.gov/gorf/gorf.html) provided by the National Center for Biotechnology Information (NCBI), accessed in April of 2013. The nucleotide sequence of the environmental nuclease characterized in this work has been deposited in the GenBank database under the Accession Number KJ436741.

The predicted ORF was used as the query in DELTA-Blast of the BLAST package (http://blast.ncbi.nlm.nih.gov/Blast.cgi) and compared with protein sequences in the non-redundant (nr) GenBank and CDD databases. The molecular weight and theoretical isoelectric point (pI) were predicted using the Compute pI/Mw tool from ExPASy (http://www.expasy.org/tools/pi_tool.html)^55^, and the prediction of subcellular localization was computed by the PSLpred method (http://www.imtech.res.in/raghava/pslpred/)^56^. The T-coffee program^57^ was used to align the deduced protein with other subfamilies of the Exo_Endo_Phos (EEP) superfamily, which was obtained by the CDD hierarchy in addition to the data obtained from DELTA-Blast. To reduce the sequences sampled, two EEP members from each subfamily were selected based on better score alignment with the metagenomic protein. Phylogenetic reconstruction was performed by the MEGA5 program^58^ using the maximum likelihood method and WAG model. Non-uniformity of evolutionary rates among sites was modeled using a discrete Gamma distribution (+G). All positions with less than 95% site coverage and redundant EEP members were eliminated. Bootstrap values were based on 1000 replicates. Myo-inositol-1(or 4)-monophosphatase sequences belonging to the IMPase-like superfamily were used as the outgroup.

### Subcloning of the metagenomic ORF

ORF-specific forward (5′ CCA TGG AAG TGC GCA TAG CGA CAT) and reverse (5′ CTC GAG TCA AGT TAG TTC CAC AAT CAC CCT) primers were designed that contain restriction sites for the *Nco*I and *Xho*I enzymes added to their 5′ regions, respectively. The PCR product was initially cloned into the pCR-Blunt vector (Invitrogen) and sequenced. Then, the ORF of interest was excised from the cloning vector with the enzymes *Nco*I and *Xho*I and subcloned into linearized pTrc99A and pHIS parallel vectors with the enzymes *Nco*I/*Sal*I and *Nco*I/*Xho*I, respectively.

### Complementation assay

Wild type- or DNA repair-deficient strains were transformed with the appropriate vector. The overnight cultures were diluted 50X in fresh LB medium supplemented with an adequate amount of the appropriate antibiotic and grown at 37 °C under constant agitation at 180 rpm to achieve an optical density (OD) at 600 nm of 0.4 when IPTG was added to the culture. The construction of the pBC and pTrc99A vectors was induced by 1 mM and 0.1 mM IPTG, respectively. To perform a survival curve, we incubated the cultures at 37 °C with shaking for an additional 40 min to obtain cells that were still in the logarithmic phase, and then challenged them with H_2_O_2_ (0–2 mM) for 30 min. The cultures were diluted with LB broth and plated onto LB agar plates, and survival was assessed by counting colonies after 24 h. To perform the drop assay, aiming to verify if ExoMeg1 was able to affect the double mutant *xth*A/*nfo* after reaching OD_600_ of 0.8, the induced cultures were diluted in LB liquid medium (10^−1^, 10^−2^, 10^−3^ and 10^−4^) and 10 μl of each dilution, as well as undiluted culture (10^0^) and were dripped in LB agar containing the appropriate antibiotic with different concentrations of H_2_O_2_ and MMS agents. The wild type and mutant strains transformed with empty vector were used as controls. The plates were incubated at 37 °C for 24 h.

GraphPad Prism version 5.00 for Windows (GraphPad Software, San Diego California USA) was used to perform the statistical analyses of survival curve. Two-way analysis of variance (ANOVA) followed by the Bonferroni post-test were performed. Difference was considered significant when P < 0.05.

### Expression of the recombinant protein

To obtain the recombinant protein, *E. coli* BL21 carrying pHis-ExoMeg1 was grown in two liters of LB medium, containing 100 μg/mL ampicillin and 34 μg/mL chloramphenicol at 37 °C. The cells were cultured to an OD_600_ of 0.45, and expression of the environmental gene was induced by adding IPTG to a final concentration of 0.1 mM and culturing for 20 h at 16 °C. The cell extract was collected by centrifugation at 15,000 g for 15 min, resuspended in PBS (pH 7.2). Lysis was accomplished by the addition of lysis buffer (0.5 M NaCl, 10% glycerol, 20 Mm Hepes pH 8.0, 5 mM imidazole and 2 mM β-mercaptoethanol), 1 mM of the protease inhibitor PMSF, 1 mg/mL lysozyme, 0.5% of 10% Triton X-100 and 150 U Benzonase nuclease (Novagen, 10 KU, 25 U/μL). After incubation on ice for 1 h, the sample was sonicated until viscosity was diminished. The soluble cell extract obtained by ultracentrifugation for 45 min at 4 °C was subjected to chromatography on a HisTrap HP column (GE Healthcare). The protein was eluted at 300 mM imidazole and was dialyzed against buffer A (0.05 M KCl, 20 mM Hepes pH 8.0, 1 mM EDTA pH 8.0, 1 mM Dithiothreitol and 10% Glycerol) using membrane SnakeSkin dialysis tubing 10,000 MWCO (Thermo Scientific) over a period of 18 h at 4 °C under agitation. The dialysate was loaded onto a Hitrap Q HP column (GE Healthcare), which was loaded with a salt gradient using Buffer A with the addition of 5, 10, 20, 30, 40, 50 and 100% buffer B (1 M KCl, 20 mM Hepes pH 8.0, 1 mM EDTA pH 8.0, 1 mM Dithiothreitol and 10% glycerol). At the end, all steps of the purification process, including the induced and uninduced samples, were applied to a 12% SDS-PAGE. The fraction containing the eluted proteins was frozen with liquid nitrogen and kept in a freezer at −80 °C.

### Substrates and activity assays

The AP endonuclease activity of environmentally purified protein was verified on single or double-stranded substrates with or without an abasic site at position 10 of the oligonucleotide 21-mer (ssDNA, ss-AP-DNA, dsDNA and ds-AP-DNA). This oligonucleotide was 5′-fluorescently labeled with Cy5. The 30-mer oligonucleotide, containing the abasic site analogue tetrahydrofuran at position 19 (Table 2), was 3′-labeled (ds-AP-DNA) with dCTP-Alexa Fluor and Terminal Transferase (Promega).

Standard reactions (20 μl) containing buffer 1 (5 mM Tris-HCl, 5 mM MgCl2, 0.5 mM dithiothreitol pH 7.0), 1 μM substrate, single (ssDNA) or double-stranded (dsDNA) DNA, and increasing concentrations of purified protein (0, 10, 20, 50 and 100 μM) were maintained at 37 °C for 1 hr. The reactions were terminated with the “STOP” solution (98% formamide and 0.5 M EDTA). The samples were heated at 95 °C for 3 min and immediately chilled on ice before applying to a PAGE-Urea gel (20% acrylamide and 8 M urea). The gels were run for 1 hr at 15 W and scanned with an FLA-5000 image analyzer (FUJIFILM) to detect the labeled DNA. Subsequently, all assays were performed using 100 μM of purified protein in each reaction, and the control reactions were run without any added protein. For time courses, the reactions were maintained at 37 °C and an aliquot of 10 μL of reaction was taken after 5, 10, 20, 30, 40, 50 and 60 min. To investigate the dependence of the divalent metal cofactor for protein activity, the reactions were performed in buffer 2 (5 mM Tris-HCl, 0.5 mM dithiothreitol pH 7.0) using increasing concentrations of MgCl2 (0, 5, 10 and 15 mM). To test whether nuclease activity can be inactivated by EDTA, we performed standard reactions with increasing concentrations of EDTA (0, 1, 5 and 10 mM). In addition, to examine whether the activity of the metagenomic nuclease may be affected by salt concentration, we performed standard reactions by adding increasing concentrations of KCl (0–1 M).

## Additional Information

**How to cite this article**: Silva-Portela, R. C. B. *et al.* ExoMeg1: a new exonuclease from metagenomic library. *Sci. Rep.* **6**, 19712; doi: 10.1038/srep19712 (2016).

## Figures and Tables

**Table 1:** Strains and plasmids used in this study.

Strain or plasmid	Genotype/Description	Reference
Strains		
DH10B	F^−^endA1 **recA1** galE15 galK16 nupGrpsLΔlacX74 Φ80lacZΔM15 araD139 Δ(ara,leu)7697 mcrAΔ(mrr-hsdRMS-mcrBC) λ^−^	51
AB1157	thr-1, araC14, leuB6(Am), Δ(gpt-proA)62, lacY1, tsx-33, qsr′-0, glnV44(AS), galK2(Oc), LAM-, Rac-0, hisG4(Oc), rfbC1, mgl-51, rpoS396(Am), rpsL31(strR), kdgK51, xylA5, mtl-1, argE3(Oc), thi-1	52
AB1157 *xth*A^−^/*nfo*^−^	As AB1157 butnfo::Kan, Δ(xthA-pncA)DE	CNRS
BL21 (DE3) pLysS	F- ompT gal dcmlonhsdSB(rB- mB-) λ(DE3) pLysS(cmR)	Promega
Plasmids	Description	
pBC SK	Cloning vector, Cam^r^	Stratagene
pBC-41 F	pBC vector plus 1.5 kb environmental insert	This study
pCR-Blunt II-TOPO	Cloning vector ,Kan^r^	Invitrogen
pCR-Blunt-EXOMEG1	pCR-Blunt vector plus 975 pb environmental ORF	This study
pHis*Parallel*	Expression vector, IPTG inducible, Amp^r^	53
His-EXOMEG1	pHis vector plus 975 pb environmental gene	This study
pTrc99A	Cloning vector, IPTG inducible, Amp^r^, it allows T7 polymerase-independent expression	54
pTrc-Exomeg1	pTrc99A plus 975 pb environmental gene	This study

**Table 2:** Labelled Oligonucleotides: -X: AP- site; THF: tetrahydrofuran.

Control (1)	5′Cy5-CGG AAT TAA AGG GCA AGA CCT-3′
AP (1)	5′Cy5-CGG AAT TAA AG_**X**_GCA AGA CCT-3′
Complementary (1)	5′ AGG TCT TGC CCT TTA ATT CCG-3′
AP (2)	5′-ATA TAC CGC GG_**THF**_ CGG CCG ATC AAG CTT ATT -AF3′
Complementary (2)	5′- AAT AAG CTT GAT CGG CCG GCC GCG GTA TAT- 3′
